# Exploring the Osteoinductive Potential of Bacterial Pyomelanin Derived from *Pseudomonas aeruginosa* in a Human Osteoblast Model

**DOI:** 10.3390/ijms252413406

**Published:** 2024-12-14

**Authors:** Mateusz M. Urbaniak, Karolina Rudnicka, Przemysław Płociński, Magdalena Chmiela

**Affiliations:** 1 Department of Immunology and Infectious Biology, Faculty of Biology and Environmental Protection, University of Lodz, 12/16 Banacha St, 90-237 Łódź, Poland; mateusz.urbaniak@edu.uni.lodz.pl (M.M.U.); karolina.rudnicka@biol.uni.lodz.pl (K.R.); przemyslaw.plocinski@biol.uni.lodz.pl (P.P.); 2 Department of Inorganic and Analytical Chemistry, Faculty of Chemistry, University of Lodz, 12 Tamka St, 91-403 Łódź, Poland

**Keywords:** pyomelanin, osteoinduction, osteoblast, bone regeneration

## Abstract

Alkaptonuria (AKU) is a genetically determined disease associated with disorders of tyrosine metabolism. In AKU, the deposition of homogentisic acid polymers contributes to the pathological ossification of cartilage tissue. The controlled use of biomimetics similar to deposits observed in cartilage during AKU potentially may serve the development of new bone regeneration therapy based on the activation of osteoblasts. The proposed biomimetic is pyomelanin (PyoM), a polymeric biomacromolecule synthesized by *Pseudomonas aeruginosa*. This work presents comprehensive data on the osteoinductive, pro-regenerative, and antibacterial properties, as well as the cytocompatibility, of water-soluble (PyoM_sol_) or water-insoluble (PyoM_insol_) PyoM. Both variants of PyoM support osteoinductive processes as well as the maturation of osteoblasts in cell cultures in vitro due to the upregulation of bone-formation markers, osteocalcin (OC), and alkaline phosphatase (ALP). Furthermore, the cytokines involved in these processes were elevated in cell cultures of osteoblasts exposed to PyoM: tumor necrosis factor (TNF)-α, interleukin (IL)-6, and IL-10. The PyoM variants are cytocompatible in a wide concentration range and limit the doxorubicin-induced apoptosis of osteoblasts. This cytoprotective PyoM activity is correlated with an increased migration of osteoblasts. Moreover, PyoM_sol_ and PyoM_insol_ exhibit antibacterial activity against staphylococci isolated from infected bones. The osteoinductive, pro-regenerative, and antiapoptotic effects achieved through PyoM stimulation prompt the development of new biocomposites modified with this bacterial biopolymer for medical use.

## 1. Introduction

Regeneration of damaged bone is a complex process involving hematopoietic cells, bone-forming cells (osteoblasts) and osteoclasts, immune cells, and endothelial cells. Bone repair consists of three primary phases: inflammation, bone production, and bone remodeling [1]. Osteoblasts derived from mesenchymal stem cells, constituting approximately 4–6% of bone cells, are involved in bone formation [2]. Mature osteoblasts synthesize extracellular type I collagen (COL1) and non-collagen proteins including osteocalcin (OC), osteopontin (OPN), and alkaline phosphatase (ALP). The extracellular matrix appears initially in the form of unmineralized osteoid, which then undergoes mineralization with the participation of hydroxyapatite and OC as well as ALP [3]. During bone remodeling, progenitor osteoblasts infiltrate the milieu of damaged bone; mature osteoclasts resorb damaged bone tissue, and then osteoclasts die, and after that, osteoblast progenitor cells are recruited; in the last step of bone formation, mature osteoblasts produce new bone osteoid [4,5].

In the physiological microenvironment of bone tissue, bone formation by osteoblasts remains in balance with the phase of bone resorption provided by osteoclasts. These opposing activities of osteoblasts and osteoclasts ensure constant bone mass and mineralization [6]. Many proteins and cytokines secreted by osteoblasts, osteoclasts, and immune cells involved at various stages of bone turnover are responsible for maintaining the balance between the process of bone formation and resorption [7]. The key modifiers responsible for maintaining the balance in bone remodeling include OC, ALP, osteoprotegerin (OPG), bone morphogenetic proteins, receptor activator of nuclear factor kappa-light-chain-enhancer of activated B cells (RANK) ligand (RANKL), interleukin (IL)-1, IL-6, IL-10, and tumor necrosis factor (TNF)-α [8,9,10]. The reasons for the bone damage can be different, such as injuries, fractures, metabolic or infectious diseases, osteoporosis, or cancer. This is a significant problem if the pathological disorders are greater than the spontaneous bone repair linked to the osteoblast’s activity [11]. The process of bone repair in connection with a fracture, osteoporosis, or implantation procedure may be even more limited if a bacterial infection accompanies it. Therefore, searching for new compounds with multidirectional pro-regenerative, osteogenic, and antibacterial activity is crucial [12,13,14].

Alkaptonuria is a metabolic disorder caused by a deficiency of homogentisate 1,2-dioxygenase in tyrosine metabolism [15]. As a consequence, homogentisic acid (HGA) does not undergo metabolic transformations, and over time, it polymerizes and accumulates in collagen tissues, which leads to ochronosis. The process of ochronotic deposit formation is not fully understood. HGA is believed to polymerize into a benzoquinone intermediate before assuming its final polymeric form in collagen tissues [15,16]. One of the most typical symptoms of AKU is chronic joint degeneration, manifested by structural changes and mineralization occurring in cartilage tissue [17]. The accumulation of HGA polymers causes hydroxyapatite deposition, a mineral responsible for bone calcification, which further hardens the connective tissue [18].

In this study, by mimicking the pathological ossification induced in AKU by HGA polymers, we aimed to support osteoinduction and osteoblasts maturation by using pyomelanin (PyoM) produced by *Pseudomonas aeruginosa*, previously shown to be cytocompatible biomimetic of ochronotic deposits. It is possible that due to the above mechanisms accompanying AKU, including the local accumulation of HGA polymers, PyoM may have proregenerative potential toward bone tissue [19,20,21]. PyoM is a dark brown-to-black negatively charged extracellular polymeric pigment of HGA produced during L-tyrosine catabolism [22]. Previously, we obtained two forms of this bacterial pigment: water-soluble (PyoM_sol_) and water-insoluble (PyoM_insol_), depending on the isolation procedure from post-cultured supernatants [23]. The chemical analysis of PyoM showed that it contains quinone and hydroxyquinone groups, which are responsible for antioxidant and reducing properties, respectively. It has been shown that due to these features, PyoM protects bacterial cells against oxidative stress and UV light, electron transport, and binding of metal ions [24,25]. We and other researchers have found that PyoM possesses antibacterial activity against various bacterial species and supports the regeneration of gastric epithelial cells in vitro by the neutralization of reactive oxygen species [23,26,27]. PyoM also stimulates the activation of the nuclear factor kappa-light-chain-enhancer of activated B cells (NF-κB), which is involved in signaling pathways driving inflammatory responses, tissue reconstruction, including bone tissue, and regeneration processes [23,28].

This study aimed to determine the cytocompatibility of PyoM_sol_ and PyoM_insol_ in vitro toward human osteoblasts in conjunction with apoptosis assessment. In addition, cell migration in a wound healing assay and cell proliferation were assessed, which are determinants of the pro-regenerative activity of cells. Furthermore, the biological activities of PyoM toward human osteoblasts were tested regarding the role of PyoM as a biomimetic polymer of ochronotic deposits in osteoinduction through an assessment of the production of OC as a unique non-collagen protein of the bone matrix produced by osteoblasts, extracellular matrix calcification, and ALP involved in osteoblast maturation. Since bone formation depends on environmental cytokines, we determined the key cytokines, IL-6, TNF-α, and IL-10, that regulate this process. In addition, the antibacterial activity of PyoM against staphylococci, the leading cause of bone infections accompanying medical interventions, was assessed by a resazurin reduction assay.

## 2. Results

### 2.1. Bacterial Pyomelanin Is a Cytocompatible Biomacromolecule Toward Human Osteoblasts and Diminishes DOX-Induced Cell Apoptosis

**Figure 1:**
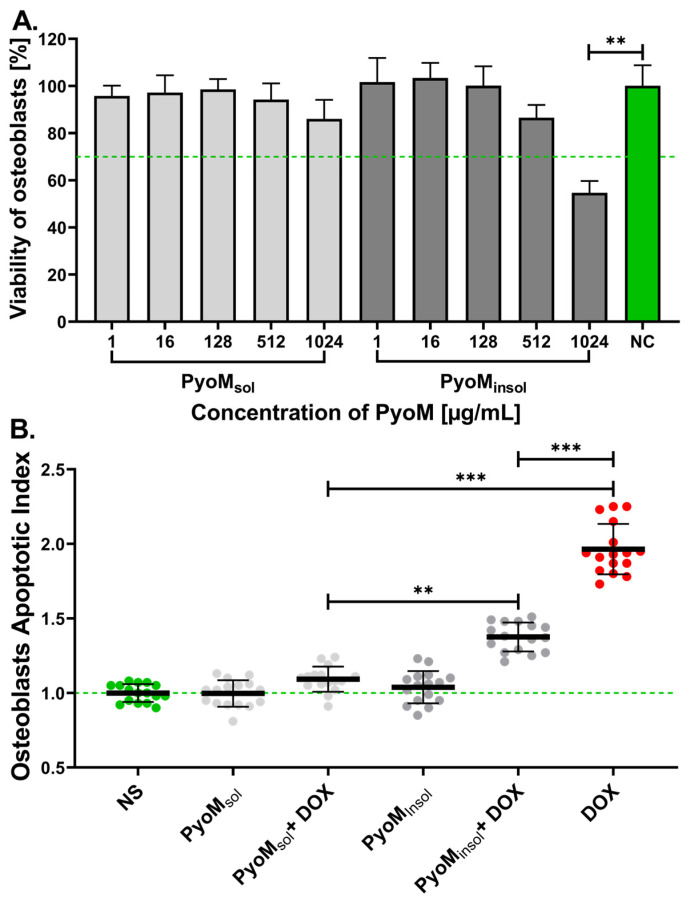
(**A**) The percentage of viable hFOB 1.19 osteoblasts after 24 h exposure to different concentrations of the water-soluble (PyoM_sol_) or water-insoluble pyomelanin (PyoM_insol_). Osteoblasts incubated only in medium (NC = 100% cell viability). (**B**) Diminishing cell apoptosis induced by doxorubicin (DOX) in the milieu of PyoM_sol_ or PyoM_insol_ at the concentration of 1 µg/mL. The Apoptotic Index was determined from the relative fluorescence units (RFUs) of cells exposed to PyoM vs. RFUs of non-stimulated osteoblasts (NS). The mean ± standard deviation results of four separate experiments are shown. Statistical significance for ** *p* < 0.01; *** *p* < 0.001.

In this study, we demonstrated that bacterial pyomelanin (PyoM_sol_ and PyoM_insol_ form) was cytocompatible toward hFOB 1.19 human osteoblasts in cell cultures in vitro in a wide concentration range (Figure 1A). PyoM_sol_ did not reduce the viability of osteoblasts in concentrations ranging from 1 μg/mL to 1024 μg/mL and met the requirements (>70.0% of viable cells) for cytocompatibility resulting from the ISO 10993-5-2009 [29] standard. A similar effect was demonstrated for PyoM_insol_; however, at a concentration of 1024 μg/mL, this PyoM variant caused a significant (*p* < 0.01) decrease up to 54.7% ± 5.0% in human osteoblast viability compared to the untreated cells (100% viable cells).

Neither PyoM_sol_ nor PyoM_insol_ alone induced cell apoptosis. There was no increase in the Apoptotic Index (AI) established for the PyoM-treated cells vs. the untreated cells (Figure 1B). The AI for hFOB 1.19 osteoblasts increased significantly (*p* < 0.001) after cell exposure to proapoptotic DOX (positive control in apoptosis assay) up to 1.96 ± 0.17 vs. AI = 1.00 for DOX untreated cells. We showed that both PyoM variants diminished the apoptotic effect of DOX. After 24 h of co-stimulation of osteoblasts with DOX and PyoM_sol_ or PyoM_insol_, the AI was significantly (*p* < 0.001) lower than in cells treated with DOX and amounted to 1.09 ± 0.08 and 1.37 ± 0.10, respectively. Moreover, PyoM_sol_ abolished the proapoptotic activity of DOX more effectively (*p* < 0.01) than PyoM_insol_.

### 2.2. Pyomelanin Promotes the Migration of Osteoblasts in Wound Healing Assay

**Figure 2:**
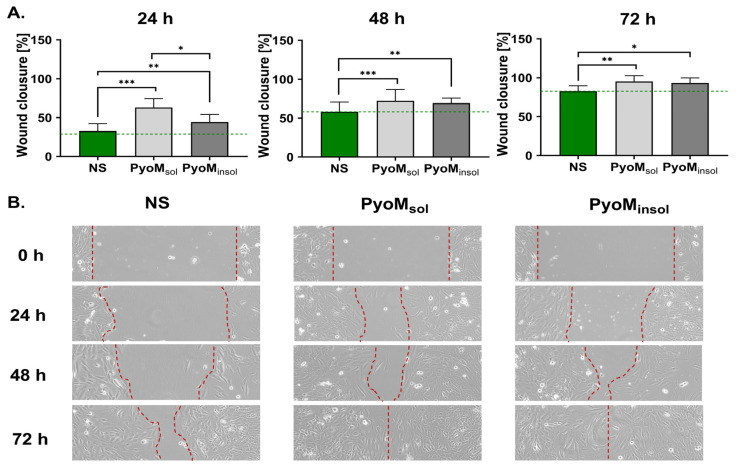
(**A**) The migration effectiveness of hFOB 1.19 osteoblasts determined in a scratch assay. Osteoblasts were cultivated for 24 h, 48 h, or 72 h in the presence of water-soluble (PyoM_sol_) or water-insoluble pyomelanin (PyoM_insol_) at a concentration of 1 µg/mL Mean ± standard deviation of five separate experiments are shown. * *p* < 0.05; ** *p* < 0.01; *** *p* < 0.001, statistically significant differences. Cells not stimulated with PyoM (NS). The reference wound closure of non-stimulated cells is marked on the graph with dashed green line. (**B**) Representative images of hFOB 1.19 osteoblast migration after 24, 48, and 72 h of water-soluble (PyoM_sol_) or water-insoluble pyomelanin (PyoM_insol_) stimulation. In the microscopic images, the red dashed lines indicate the width of the overgrown crack.

Migration of osteoblasts was significantly enhanced in the wound area after 24, 48, and 72 h, and the effectiveness of wound closure was 63.1% ± 9.2%, 72.2% ± 11.7%, and 95.1% ± 7.6%, respectively (Figure 2A). Similar results were shown for osteoblasts exposed to PyoM_insol_. PyoM_insol_, at the same time points as PyoM, significantly promoted the cell migration by up to 44.7% ± 9.6%, 69.4% ± 6.4%, and 93.2% ± 6.7% confluence, respectively. Only after 24 h of exposure to PyoM_sol_ did the cells migrate more effectively (*p* < 0.05) than the cells treated with PyoM_insol_. The migration of untreated hFOB 1.19 osteoblasts in the scratched area was 32.7% ± 9.5%, 58.0% ± 10.6%, and 82.8% ± 6.7% after 24, 48, and 72 h, respectively. Representative images of hFOB 1.19 osteoblast migration after 24, 48, and 72 h of PyoM stimulation are shown in Figure 2B.

### 2.3. Pyomelanin Promotes Maturation of Human Osteoblasts

**Figure 3:**
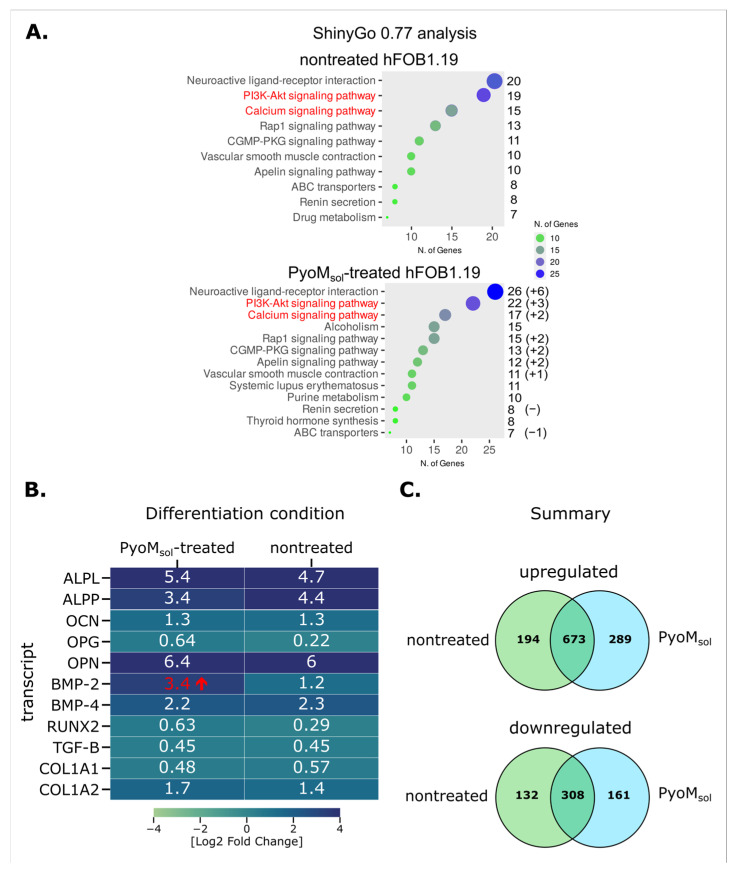
Transcriptomic changes in hFOB 1.19 osteoblasts upon treatment with soluble pyomelanin (PyoM_sol_). (**A**) Ontology analysis of differentially expressed genes overexpressed in hFOB1.19 osteoblasts during differentiation in osteoinductive media utilizing the ShinyGo 0.77 online platform [30]. (**B**) List of transcripts relevant to osteoblastic differentiation of hFOB cells in comparison between proliferative and differentiation conditions for PyoM_sol_-treated and non-treated cells. (**C**) A summary of transcriptomic changes observed between hFOB 1.19 cells differentiating under standard conditions versus cells differentiating in media supplemented with PyoM_sol_. RNA was isolated from cells incubated in proliferative or differentiation conditions for 14 days. The change in BMP-2 expression between non-treated and treated cells (marked in red) exceeded the Log 2 FC of 2, preset as the threshold for our analysis. ALPL, liver-/bone-/kidney-specific or tissue-nonspecific (TNSALP) ALP form, ALPP, placental ALP form; BMP, bone morphogenic protein; CGMP-PKG, cyclic guanosine monophosphate protein kinase G; COL, collagen; OCN, osteocalcin; OPG, osteoprotegrin, RUNX, runt-related transcription factors; TGF-Beta, transforming growth factor beta; PI3-Akt, PI3-Akt; phosphatidylinositol 3-kinase, serine/threonine kinase (protein kinase B); Rap1, Ras-proximate-1 or Ras-related protein-1.

Transcriptomic analyses revealed that the PyoM_sol_-treated osteoblasts were activated compared to the untreated cells, as shown by the number of differentially expressed transcripts. The non-treated cells revealed 1307 transcriptional changes, and the PyoM_sol_-treated cells exhibited 1431 significant changes (Table S1). Relative changes in expression were also more pronounced in the case of the pyomelanin-treated cells. A selection of transcripts relevant to osteoblast activation are presented in Figure 3. Transcriptomic changes associated specifically with hFOB 1.19 activation and differentiation upon PyoM_sol_ treatment include a significant upregulation of bone morphogenic protein (BMP)-2 and a tendency to an increased expression of liver-/bone-/kidney-specific or tissue-nonspecific (TNSALP) form of alkaline phosphatase related to the bone maturation, in conjunction with the upregulation of phosphatidylinositol 3-kinase–serine/threonine kinase (PI3K-Akt) and calcium-dependent signaling pathways involved in cell proliferation and differentiation. The upregulation of the above proteins in the osteoblasts exposed to PyoM_sol_ confirms that PyoM may play a role in osteoinduction.

**Figure 4:**
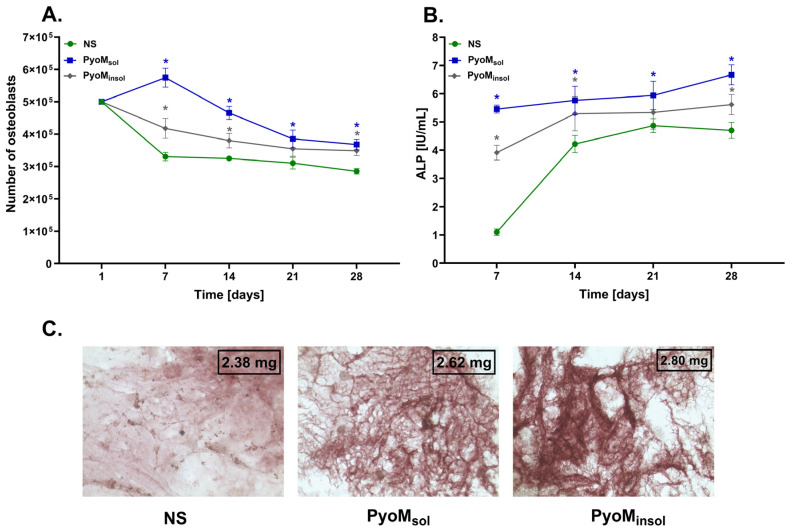
The influence of pyomelanin (PyoM) on cell growth, alkaline phosphatase production, and bone cell calcification. (**A**) Number of hFOB 1.19 osteoblasts after 1, 7, 14, 21, or 28 days of incubation with water-soluble pyomelanin (PyoM_sol_), water-insoluble pyomelanin (PyoM_insol_), or culture medium alone, i.e., unstimulated cells (NS). (**B**) The activity of alkaline phosphatase (ALP) produced by hFOB 1.19 osteoblasts in cell cultures exposed to PyoM_sol_, PyoM_insol_, or culture medium alone (NS) after 7, 14, 21, or 28 days are shown in international units (IU). Results are shown as mean ± standard deviation. The experiment was performed four times. Statistical significance is indicated by * at *p* < 0.05. (**C**) Representative images of calcification process in cell culture of osteoblasts exposed for 24 days to PyoM_sol_ or PyoM_insol_ or not stimulated (NS). The cells were stained with 4% alizarin The stained mineralized extracellular matrix of osteoblasts was observed under an inverted-phase contrast microscope. Calcium deposits were assessed quantitatively on the basis of absorbance values at 405 nm using a standard curve developed with hydroxyapatite.

The assessment of cell proliferation was made based on DNA quantification using a fluorescence assay, in which the fluorescence intensity of DNA corresponds to the number of cells. In the cell cultures stimulated with PyoM_sol_ or PyoM_insol_, the number of cells was higher within the cultivation period (*p* < 0.05) compared to the unstimulated cells (Figure 4A). The mean number of cells after 28 days of stimulation with either PyoM_sol_ (3.67 × 10^5^ ± 0.16 × 10^5^) or PyoM_insol_ (3.49 × 10^5^ ± 0.15 × 10^5^) was significantly higher (*p* < 0.05) than the number of cells in the untreated cultures (2.85 × 10^5^ ± 0.09 × 10^5^). PyoM_sol_ promoted efficient osteoblast proliferation on the 7th day of osteogenic culture compared to the cells on the first day of the experiment.

Moreover, the stimulation of the osteoblasts with PyoM_sol_ or PyoM_insol_ led to a significant increase in ALP production (Figure 4B). On the 28th day of culture, the ALP concentration reached 6.67 ± 0.35 IU/mL, 5.61 ± 0.26 IU/mL, and 4.70 ± 0.29 IU/mL after 7, 14, 21, and 28 days of culture, respectively (*p* < 0.05).

**Figure 5:**
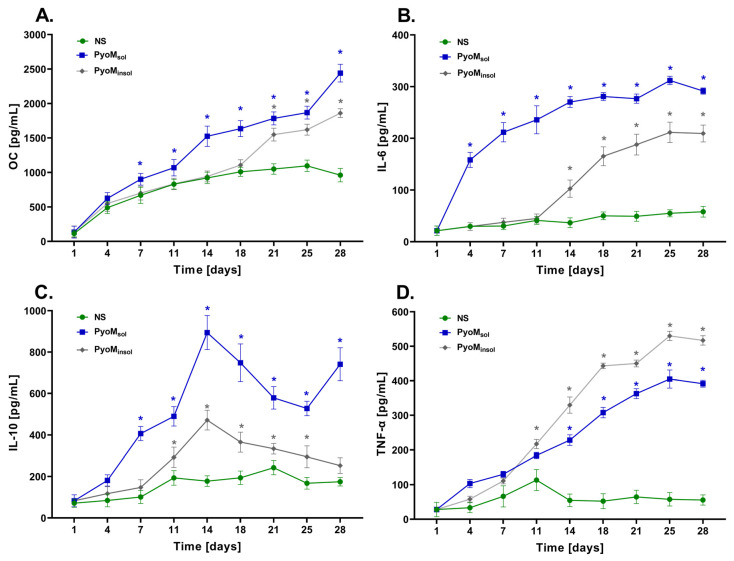
Secretion of osteocalcin and cytokines by osteoblasts exposed to PyoM. (**A**) The levels of osteocalcin (OC), (**B**) the levels of interleukin (IL)-6, (**C**) the levels of IL-10, and (**D**) the levels of tumor necrosis factor (TNF)-α in cell cultures of hFOB 1.19 exposed to water-soluble pyomelanin (PyoM_sol_), water-insoluble pyomelanin (PyoM_insol_), or culture medium alone, i.e., non-stimulated cells (NS), after 1, 4, 7, 11, 14, 18, 21, 25, and 28 days. Results are shown as mean ± standard deviation. The experiment was performed four times. * *p* < 0.05 indicates statistically significant differences.

The secretion of osteocalcin (OC) by the osteoblasts, the specific biomarker of osteogenesis, increased significantly in the supernatants of the culture of hFOB 1.19 osteoblasts exposed to PyoM_sol_ throughout the entire culture period (Figure 5A), with the highest level observed on the 28th day. The level of OC on the 7th day increased up to 901.0 ± 85.3 pg/mL or 668.1 ± 117.7 pg/mL and 2440.2 ± 128.3 pg/mL or 961.2 ± 97.7 pg/mL on 28th day, respectively. Significant differences (*p* < 0.05) in OC secretion were also observed in the cell cultures containing PyoM_insol_ vs. the unstimulated cell cultures, but only on the 21st day of culture.

The elevated levels of ALP and OC in the cell cultures of osteoblasts in the milieu of PyoM_sol_ or PyoM_insol_ (Figure 4B and Figure 5A) were associated with an increased calcification process (Figure 4C).

As shown in Figure 5B, the production of pleiotropic interleukin IL-6, which is involved in bone formation, increased significantly (*p* < 0.05) throughout the entire course of the experiment in the osteoblasts culture treated with PyoM_sol_, reaching 291.5 ± 6.0 pg/mL on day 28, compared to the unstimulated cultures (58.0 ± 10.4 pg/mL). The increasing trend in IL-6 secretion was also demonstrated for the cell cultures treated with PyoM_insol_; however, the difference was statistically significant from the 14th day of culture.

A similar secretion profile was found for IL-10, which has regulatory activity in the immune system and also promotes osteogenesis. The maximum concentrations of IL-10 in the hFOB 1.19 osteoblast cultures exposed to PyoM_sol_ or PyoM_insol_ was demonstrated on the 14th day of the experiment, and they were 849.1 ± 82.3 pg/mL and 471.2 ± 47.5 pg/mL, respectively (Figure 5C). Then, the concentration of IL-10 decreased over time in the cell cultures exposed to PyoM_insol_, while in the cell cultures treated with PyoM_sol_, there was a second peak of IL-10 elevation from 25th day to the 28th day of culture. At the endpoint, on the 28th day, the concentration of IL-10 in the osteoblast culture supernatant after stimulation of the cells with PyoM_sol_ or PyoM_insol_ was significantly higher (*p* < 0.05) compared to the culture of osteoblasts propagated in the culture medium alone, amounting to 740.9 ± 79.8 pg/mL, 252.6 ± 37.7 pg/mL, and 174.6 ± 20.5 pg/mL, respectively.

The level of TNF-α was significantly (*p* < 0.05) higher in the cell culture supernatants after stimulation of the cells with PyoM_sol_ or PyoM_insol_ on the 28th day, amounting to 391.2 ± 28.2 pg/mL and 517.0 ± 38.5 pg/mL, respectively, compared to the unstimulated osteoblasts, at 55.5 ± 14.7 pg/mL (Figure 5D).

### 2.4. Antibacterial Activity of PyoM Toward Staphylococcus spp.

**Figure 6:**
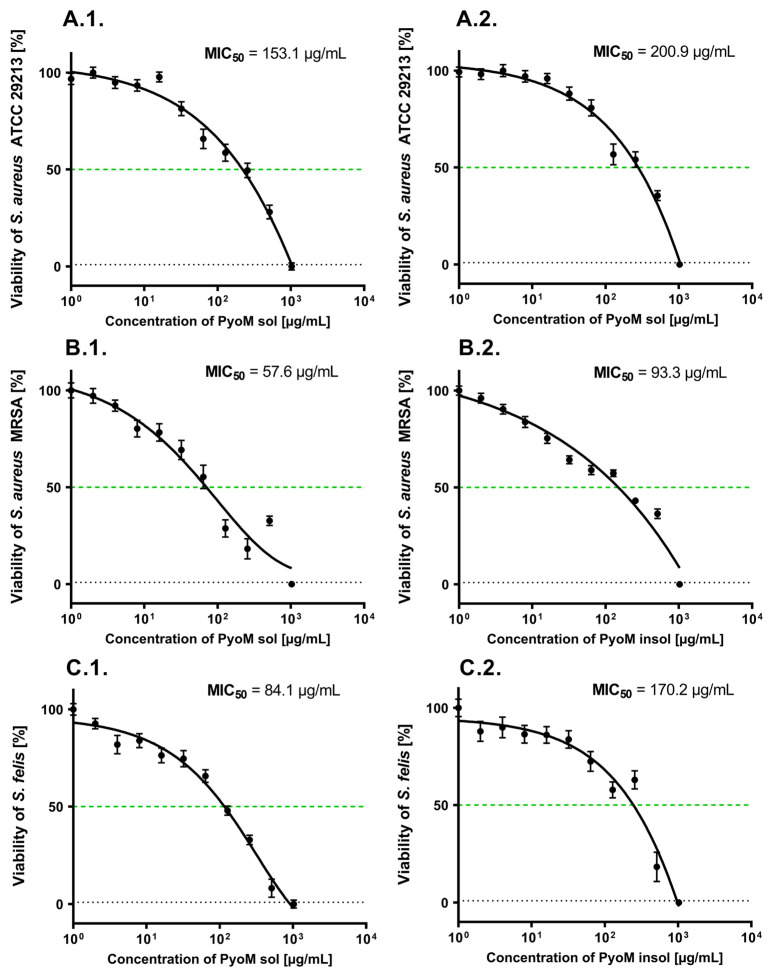
Antibacterial activity of studied PyoM variants. The figure shows dose–response curves supplemented with the minimum inhibitory concentration (MIC)_50_ determined for (**1**) water-soluble piomelanin (PyoM_sol_) or (**2**) water-insoluble pyomelanin (PyoM_insol_). *Staphylococcus* strains: (**A**) reference *S. aureus* ATTC 29213, (**B**) clinical *S. aureus* strain resistant to methicillin (MRSA), and (**C**) *S. felis*. Results are shown as mean ± standard deviation. The experiment was performed five times. The green dashed line shows the 50% bacterial viability.

The dose–response curves and MIC_50_ of PyoM against the reference *S. aureus* ATCC 29,213 and two clinical isolates (*S. aureus* MRSA and *S. felis*) are shown in Figure 6. The MIC_50_ was defined as the lowest concentration of bacterial PyoM at which 50% of the bacterial cells were killed. Both forms of PyoM significantly (*p* < 0.001) reduced the viability of the reference and clinical strains of *Staphylococcus* spp., as examined by the resazurin reduction assay. The lowest MIC_50_ (57.6 μg/mL) for PyoM_sol_ was observed for the clinical *S. aureus* MRSA strain, while the highest MIC_50_ (153.1 μg/mL) was identified for the reference *S. aureus* ATCC 29213. A similar inhibition of metabolic activity was shown for PyoM_insol_, where the lowest MIC_50_ (93.3 μg/mL) was obtained for *S. aureus* MRSA, and the highest MIC_50_ (200.9 μg/mL) was demonstrated for *S. aureus* ATCC 29213. PyoM_sol_ effectively limited the metabolism of the tested bacteria compared to PyoM_insol_, which resulted in a significantly (*p* < 0.001) lower MIC_50_.

## 3. Discussion

Bone is a dynamic tissue that undergoes continuous remodeling. This process includes bone formation followed by bone resorption, involving both osteoblasts and osteoclasts, respectively. Injured bone tissue can self-repair, restoring the damaged part to its original structure and mechanical strength [31,32] Data from 2019 show that there were 178 million bone fractures, which is 33.4% more than the number in 1990 [33]. The increasing number of bone fractures, including those related to osteoporosis, and the observed trends in the rising costs of treating these diseases have prompted researchers to look for new substances that stimulate the regeneration of bone tissue that can be used individually or as part of a biocomposite [34,35,36]. Polymers of HGA, responsible for the excessive ossification in AKU, and pyomelanin, which is a biomimetic of ochronotic deposits produced by *Pseudomonas aeruginosa*, could be good candidates [19,20,21].

This study demonstrated that PyoM_sol_ and PyoM_insol_ are cytocompatible biopolymers for human hFOB 1.19 osteoblast cells in a wide range of concentrations. In previous studies, we showed that PyoM_sol_ and PyoM_insol_ do not reduce the cell viability of reference L-929 mice fibroblasts, human THP-1 monocytes, and human AGS gastric epithelial cells [23,26]. Ferraz et. revealed that PyoM isolated from the *Pseudomonas putida* bacteria is not toxic to human A-375 skin epithelial cells, Hep G2 liver epithelial-like cells, and Caco-2 colorectal epithelial cells [37]. Moreover, PyoM isolated from *Halomonas titanicae* and *Pseudomonas stutzeri* showed a high level of cytocompatibility with human HaCeT keratinocytes and L-929 fibroblasts, respectively [24,38]. Our previous in vitro studies on the insect *Galleria mellonella* larvae model also confirmed the biocompatibility of both forms of PyoM isolated from *P. aeruginosa* [23].

In this study, PyoM_sol_ and PyoM_insol_ did not induce apoptosis in human osteoblasts and partially limited DOX-induced programmed cell death in these cells. Importantly, co-stimulation of osteoblasts with PyoM_sol_ led to a complete abolition of DOX-induced apoptosis. These data are consistent with the results obtained using gastric epithelial cells, AGS, co-stimulated with PyoM and lipopolysaccharide (LPS) *Escherichia coli* or *Helicobacter pylori*, where PyoM reversed LPS-induced apoptosis [26]. The potential anti-apoptotic effect of PyoM may reflect the anti-oxidative properties of this bacterial polymer [24]. PyoM-mediated neutralization of reactive oxygen species (ROS), increasing the frequency of DNA breaks, may prevent apoptotic cell death [26]. This antioxidant effect of PyoM in the environment where tissue regenerative processes occur, including bone tissue repair, may be particularly beneficial. The place where tissue damage occurs is infiltrated by immunocompetent inflammatory cells, which, when activated, secrete cytokines needed in the regeneration processes, but these cells also deliver ROS and proteolytic enzymes, which, in excess, promote tissue damage. Therefore, control over oxidative stress in the repair environment is necessary.

Osteoblasts are involved in bone formation and remodeling of bone tissue. Osteoblast progenitor cells undergo proliferation and secrete a bone matrix, which is then mineralized [39]. Proliferation of progenitor cells is correlated with the production of collagen, fibronectin, and OPN and a higher deposition of transforming growth factor-β (TGF-β) receptor 1. Later on, cell proliferation is diminished, and the cells enter the maturation step, where they secrete COL1 and produce ALP to mature the extracellular matrix. The final step in bone development is matrix mineralization, which is related to the production of OPN, OC, and bone sialoprotein, with the continued production of ALP and COL1 [39]. The development of bone tissue by osteoblasts and its remodeling by osteocytes are under the control of cytokines, including IL-6, TNF-α, or IL-10 and TGF-β [9,39].

Cell migration is an early marker of regeneration processes, followed by the ability of cells to proliferate. In this study, we showed that both PyoM variants promoted the migration of osteoblasts in a wound healing assay. The effectiveness of bone tissue development and its remodeling depend on progenitor cell replication effectiveness and mature osteoblast lifespan [40]. In this study, we showed that the number of cells exposed for 28 days to PyoM_sol_ or PyoM_insol_ was higher than the number of cells in cultures without PyoM. Interestingly, PyoM_sol_ on the 7th day of culture promoted osteoblast proliferation. These results suggest that PyoM_sol_ could potentially play a role of an osteoblast-growth-like factor, and both PyoM_sol_ and PyoM_insol_ are able to drive cell maturation. It has been shown in transctiptomic studies that the upregulation of PI3-Akt and calcium-channel-related signaling pathways are involved in bone cell maturation [41,42,43]. The preliminary transcriptomic analysis performed in this study showed that in the milieu of PyoM, there was an increase in transcripts of BMP-2 and a tendency to an increased expression of TNSALP related to bone maturation and the upregulation of PI3K-Akt and calcium-dependent signaling pathways involved in cell proliferation and differentiation.

In this study, we selected ALP and OC as markers of osteoblast activation and maturation in conjunction with an assessment of calcification to determine whether the studied PyoM variants could increase the osteogenic activity of osteoblasts. ALP is an extracellular membrane-bound enzyme that hydrolyzes the ester of monophosphate at an alkaline pH that is highly expressed in the cells of mineralized tissue [44]. In bones, ALP is an early marker of osteoblast activity and bone formation, and it plays an essential role in bone mineralization [45]. This study revealed that PyoM_sol_ and PyoM_insol_ led to a significant increase in ALP activity in cell cultures of osteoblasts.

OC, a non-collagen bone matrix protein, is specific for osteoblasts [46]. It has been shown that OC, which is secreted into the bone microenvironment, binds to calcium ions in hydroxyapatite and also provides mechanical bone strength due to complexing with collagen via OPN [47]. We showed that bacterial PyoM significantly increased the secretion of OC by osteoblasts in cell cultures in vitro. The elevated production of ALP and OC by osteoblasts exposed to PyoM was related to an increased calcification process, as shown by the number of calcium deposits in the cell cultures, which supports the suggestion about the pro-regenerative activity of PyoM toward bone cells.

In vivo, bone development and remodeling are facilitated by different cytokines produced by osteoblasts and inflammatory cells, including neutrophils, macrophages, and T lymphocytes [9]. IL-6 is involved in the differentiation of osteoblast precursors and protecting osteoblasts from apoptosis [48,49]. In response to IL-6, osteoblasts produce ALP, OC, and bone sialoprotein, resulting in the development of bone nodule formation and bone matrix mineralization [50]. This cytokine protects bone tissue against elevated resorption by osteoclasts by diminishing the expression of receptor activator for nuclear factor κB ligand (RANKL) in this set of cells and by increasing the production of IL-10 and IL-4, which downregulate the activity of osteoclasts [51]. In this study, we demonstrated that PyoM_sol_ and PyoM_insol_ significantly stimulated osteoblasts to secrete IL-6, although in the presence of PyoM_sol_, the levels of IL-6 were higher. The higher number of viable osteoblasts during cultivation in osteoconduction conditions and the more effective neutralization of DOX-induced apoptosis in the presence of PyoM_sol_ may be dependent on a higher level of IL-6 in these cell cultures.

Our results indicate that the stimulation of human osteoblasts with bacterial PyoM_sol_ or PyoM_insol_ resulted in a significantly increased production of IL-10, which indirectly induces bone formation through the p38 mitogen-activated protein kinase (MAPK) signaling pathway [52]. Moreover, IL-10 inhibits the differentiation of osteoclasts in the early steps of this process by diminishing RANKL expression and the expression of nuclear factor of activated T-cells (NFATC1) [53,54,55]. Animal experiments have proven that IL-10-deficient mice exhibit reduced bone mass, increased mechanical fragility, and inhibition of bone formation [56,57]. Bacterial PyoM may potentially be beneficial in controlling excessive osteoclastogenesis through IL-10.

TNF-α induces bone resorption and promotes osteoclast formation from bone-marrow-derived macrophages as well as osteoclast differentiation [58]. TNF-α initiates and activates osteoclasts to resorb bone, which may benefit bone repair during bone healing. It has been shown that this cytokine, in low concentrations, stimulates the differentiation of osteoblasts from mesenchymal precursors. However, in high concentrations, TNF-α inhibits bone formation [59,60]. In this study, the concentration of TNF-α in the cultures of osteoblasts exposed to the tested PyoM variants was higher than in the control cell cultures. The level of TNF-α was higher after the treatment of the cells with PyoM_insol_ than after treating them with PyoM_sol_. This may have been a result of different ligand–TNF-α receptor interactions; however, the explanation of this suggestion needs further study. The observed elevated TNF-α secretion in response to PyoM may suggest a role of this cytokine in the development of bone tissue and/or remodeling. Considering the role of TNF-α in driving the inflammatory response, the lower activity of PyoM_sol_ compared to PyoM_insol_ in TNF-α induction may be beneficial for the bone-formation process. Although our data indicate that PyoM influenced the production of the studied cytokines by osteoblasts, at this stage of research, it is difficult to explain why the osteoblasts responded with a stronger production of IL-6 in the environment of PyoM_sol_ compared to that of PyoM_insol_. Further studies are also necessary to deepen the knowledge about the possible mechanisms differentiating the PyoM variants as TNF-α stimulators and to confirm the role of PyoM in driving the production of IL-10 and its role in the regulation of osteogenesis.

The increasing number of antibiotic-resistant bacterial species has become a significant problem worldwide in medical practice. This situation requires the development of new substances with antibacterial potential that can be part of new therapies, including the usage of biocomposites for bone regeneration, reducing the risk of infection [61]. In this study, PyoM_sol_ and PyoM_insol_ exhibited antibacterial activities towards clinical *Staphylococcus* strains isolated from bone infections. The bacterial killing was more effective after treatment of the tested strains with PyoM_sol_ than with PyoM_insol_. Other researchers have also demonstrated the antibacterial and antifungal properties of PyoM. For example, melanin from *Pseudomonas balearica* successfully reduced the viability of pathogenic *S. aureus*, *E. coli*, and *Candida albicans* [62]. Xu et al., based on experiments with *Vibrio parahaemolyticus* and *S. aureus*, suggested that PyoM damages bacterial cell membranes [63]. PyoM has also been shown to inhibit the growth of *H. pylori* [26,64].

This work is a starting point to elucidate the antiapoptotic, proregenerative, and osteoinductive mechanisms of PyoM, providing insight into the characteristics of osteoblast maturation marker activity. PyoM, unlike HGA, increased ALP activity, did not induce a cytotoxic effect, and supports osteoblast proliferation [65]. However, further molecular studies are required to determine the mechanism by which bacterial PyoM promotes osteoblast maturation. The effects of PyoM toward osteoclasts should be investigated to determine whether PyoM does not affect osteoblast–osteoclast homeostasis.

The effectiveness of PyoM as a proregenerative and osteoinductive biocomponent should be further confirmed in a dedicated animal model. Our preliminary study confirmed the biocompatibility of PyoM in a mice model, thus prompting further experiments in vivo.

## 4. Materials and Methods

### 4.1. Growth Conditions of Pseudomonas aeruginosa

The growth conditions of *P. aeruginosa* were established as previously described [23,26]. Briefly, *P. aeruginosa* Mel+ strain (Collection of Department of Immunology and Infectious Biology, University of Lodz, Poland) was inoculated after thawing onto a Luria Broth (LB) agar plate and incubated for 24 h in a microbiological incubator (37 °C). Next, the single colony was isolated and incubated in liquid LB (anaerobic conditions, 37 °C, 18 h) until log-phase. Pyomelanin Minimal Medium II (PMM II) (patent application number: PL438865) was used to increase the PyoM production [23,26]. PMM II was inoculated with 1.0 mL of bacterial suspension (1.0 McFarland scale) and incubated for 5 days (37 °C, 120 rpm). The cultures were transferred to room-temperature conditions after the appearance of a black color and exposed to sunlight to stimulate the PyoM production of the pigment.

### 4.2. Isolation and Purification of PyoM_sol_ and PyoM_insol_

Water-soluble pyomelanin (PyoM_sol_) and water-insoluble pyomelanin (PyoM_insol_) were obtained as previously described [23,26]. To isolate the PyoM_insol_, the supernatant from a centrifuged culture of *P. aeruginosa* was acidified with 6.0 M HCl (PolAura, Dywity, Poland) up to pH 2.0. After washing the PyoM_insol_ pellet with HCl and twice with distilled water, the PyoM_insol_ pellet was suspended in ethanol (Chempur, Piekary Śląskie, Poland) and then heated in a water bath (95 °C, 30 min.). The PyoM_insol_ was washed with ethanol (2×) and air-dried. The bacterial cell culture supernatant was incubated with chloroform (ratio 1:1) for 24 h (room temperature, shaking at 120 rpm) to obtain the PyoM_sol_. A separating funnel was used to separate the aqueous phase. The protein contaminants were removed by centrifugation, and then the PyoM_sol_ was purified by ultrafiltration (MWCO: 30 kDa) (Sartorius, Göttingen, Germany). The PyoM_sol_ was dried overnight at 50 °C. Affinity chromatography was performed to remove the endotoxin (Pierce™ High-Capacity Endotoxin Removal Spin Columns, Thermo Scientific, Waltham, MA, USA). The PyoM_insol_ and PyoM_sol_ were stored at 4 °C in the dark after washing the pellets with chloroform, ethyl acetate, ethanol, and water.

### 4.3. Osteoblast Growth Conditions

The hFOB 1.19 osteoblasts were cultured in DMEM/Nutrient Mixture F-12 Ham (Gibco, Zug, Switzerland) with the addition of bovine serum (heat-inactivated) and geneticin (0.3 mg/mL; Gibco, Zug, Switzerland) in the conditions of an incubator (33 °C, 5% CO_2_), as previously described [66]. For the test, the cell suspension (1 mL, 5 × 10^5^/mL) was added to a 6-well cell culture plate (Thermo Fisher Scientific, Waltham, MA, USA). Following the initial overnight incubation (cell adhesion), the medium was removed, and the control wells were completed with 1.5 mL of osteogenic medium (DMEM/Nutrient Mixture F-12 Ham), with 1% heat-inactivated fetal bovine serum, 0.3 mg/mL geneticin (Gibco, Zug, Switzerland), 50 μg/mL ascorbate-2-phosphate (Sigma Aldrich, Darmstadt, Germany), 1 μM dexamethasone (Sigma Aldrich, Darmstadt, Germany), and 10 mM β-glycerophosphate (Sigma Aldrich, Darmstadt, Germany). The test wells were supplemented with 1.5 mL of PyoM_sol_ or PyoM_insol_ (1 μg/mL) dissolved in osteogenic medium. Next, the osteoblasts were cultured at 39 °C with 5% CO_2_, and the osteogenic medium with or without the PyoM variants was changed in a three-day cycle. The supernatant samples were collected after 4, 7, 11, 14, 18, 21, 24, and 28 days and stored at −80 °C. These samples were then used for evaluation of the osteogenic markers. Cell lysates were collected on 7, 14, 21, and 28 days for the proliferation assessment and alkaline phosphatase (ALP) activity quantification, while the cell culture supernatants were collected to evaluate the level of the selected cytokines.

### 4.4. Determination of PyoM_sol_ and PyoM_insol_ Cytocompatibility Towards Osteoblasts

Studies on the cytocompatibility of PyoM_sol_ and PyoM_insol_ were carried out using human fetal hFOB 1.19 osteoblasts (ATCC, Rockville, MD, USA), as indicated in the ISO-10993-5-2009 [29] standard. The viability of the untreated cells and the cells treated with the PyoM variants was determined on the basis of the cells’ ability to reduce 3-(4,5-dimethylthiazol-2-yl)-2,5-diphenyltetrazolium bromide salt (MTT, Sigma Aldrich, Saint Louis, MO, USA) [23,66]. We used cells cultured in DMEM/Nutrient Mixture F-12 Ham (Gibco, Zug, Switzerland) with bovine serum (heat-inactivated) and geneticin (0.3 mg/mL; Gibco, Zug, Switzerland) at 33 °C with 5% CO_2_ and >90% humidity. The cell suspension (100 μL, 4 × 10^5^/mL) was added to a 96-well cell culture plate and incubated overnight as per the above procedure to receive adherent cells. The solutions of the tested PyoM_sol_ or PyoM_insol_ were added to the wells (6 repetitions). After 24 h of incubation, the cell monolayers were checked under an inverted contrast-phase microscope. The control cell cultures (in medium without PyoM) consisted of a positive control (PC) with 100% cell viability. Next, 20 μL of MTT (5 mg/mL) was added to the wells, and the plates were incubated for 4 h and then centrifuged (1200 rpm, 10 min). The supernatants were replaced with 100 mL of DMSO to dissolve the formazan crystals. After dissolving the formazan crystals, the absorbance was measured at 570 nm (Multiskan EX, ThermoFisher, Waltham, MA, USA).

### 4.5. The Assessment of Cells Undergoing Apoptosis

Cells undergoing apoptosis were identified using a commercial terminal deoxynucleotidyl transferase dUTP nick-end labeling (TUNEL) assay (Cell Meter TUNEL Apoptosis Assay Kit, AAT Bioques, Sunnyvale, CA, USA), as previously described [26]. After 24 h, the hFOB 1.19 osteoblasts that were treated with PyoM_sol_ or PyoM_insol_ (1 μg/mL) were stained fluorescently. The fluorescent dye facilitated the detection of the cells undergoing apoptosis by targeting the nicks in their DNA. Doxorubicin (DOX, Sigma Aldrich, Darmstadt, Germany) at a concentration of 50 μM was used as a positive control for the induction of osteoblast apoptosis. The level of osteoblast apoptosis exposed simultaneously to the PyoM variants and DOX was also verified. The fluorescence intensity of the apoptotic cells was measured (SpectraMax^®^ i3x Multi-Mode Microplate Reader, Molecular Devices, San Jose, CA, USA) at 550 nm (excitation) and 590 nm (emission). The relative fluorescence units (RFUs) of the PyoM-stimulated cells vs. the RFUs of the cells not stimulated with PyoM constituted the Apoptotic Index.

### 4.6. Assessment of Cell Migration in “Wound Healing Assay–Scratch Assay”

The migration of the hFOB 1.19 osteoblasts was determined using a “wound healing assay–scratch assay”, according to a previously described procedure [26,67]. Cells were added to six-well plates (5 × 10^5^ cells per well) in DMEM/Nutrient Mixture F-12 Ham (Gibco, Zug, Switzerland) supplemented with bovine serum (heat-inactivated) and geneticin (0.3 mg/mL; Gibco, Zug, Switzerland) and were incubated (33 °C, 5% CO_2_) to obtain confluent cell monolayers, which were then scratched. Widening of the scratch was treated as the beginning (time: 0 h) of wound repair. The culture medium was removed, and PyoM_sol_ or PyoM_insol_ solutions were added. Wound images at 0, 24, 48, and 72 h were registered with a digital camera in an inverted contrast-phase microscope (Motic AE2000, Xiamen, China). The wound width was measured using the software Motic AE (version Motic Images Plus 2.0 ML, Xiamen, China). The effectiveness of wound healing in the presence of PyoM was determined as the percentage of wound closure compared to wound closure in the cell cultures with untreated cells.

### 4.7. Osteoblast Proliferation

A CyQUANT cell proliferation assay (Invivogen, San Diego, CA, USA), based on the measurement of fluorescence, was used to assess the cell proliferation, as previously described [66,68]. Osteoblasts after 7, 14, 21, and 28 days of exposure to PyoM were washed with phosphate-buffered saline (PBS) and frozen at −80 °C. To assess the proliferation, the cells were thawed and lysed in a buffer containing CyQUANT-GR fluorescent dye, as recommended by the manufacturer. The fluorescence was measured at 480 nm (excitation) and 520 nm (emission) using a SpectraMax i3x Multi-Mode Microplate Reader (Molecular Devices, San Jose, CA, USA). Quantifying the cell proliferation was performed using a standard curve based on a known cell number.

### 4.8. Whole-Cell Transcriptomic Analysis by RNA Sequencing

hFOB 1.19 cells grown at 33 °C (proliferative conditions) or at 39 °C (osteoinductive conditions) were subjected to lysis using RNAse-free water mixed with TRIzol LS Reagent (Thermo Fisher Scientific, Waltham, MA, USA). RNA was isolated using Direct-zol™ RNA Miniprep Plus (Zymo Research, CA, USA), as recommended by the manufacturer. DNA contamination was removed using a TURBO DNA-free™ Kit (Thermo Fisher Scientific, Waltham, MA, USA). The RNA quantity was evaluated using Agilent RNA 6000 Nano Kit Agilent 2100 BioAnalyzer (Agilent Technologies, Santa Clara, CA, USA).

The preparation of total RNA sequencing libraries was performed following the protocol described in Kawka et al., 2023 [69]. Isolation of RNA, development of the library, and RNA sequencing were conducted three times. Purified RNA beads (2 µg) (AMPure XP, Becton Dickinson, Burlington, NC, USA) underwent rRNA depletion using a Ribo-off rRNA Depletion Kit (Human/Mouse/Rat, Vazyme, Nanjing, China). The sequencing libraries were then prepared using a KAPA Stranded RNA-Seq Kit (KAPA Biosystems, Roche, Basel, Switzerland). The quantity and quality of the libraries were assessed using an Agilent 2100 BioAnalyzer with a DNA 1000 chip. Subsequently, the cDNA libraries were sequenced using a NextSeq 550 system with a NextSeq 500/550 Mid Output v.2 Sequencing Kit (150 cycles, 2 × 75 bp) (Illumina, San Diego, CA, USA), ensuring a yield of 5 to 10 million paired-end reads per sample library. For the analysis of RNA-Seq data, Cutadapt version 2.8 was exploited to eliminate sequencing adapters, followed by quality trimming using Sickle version 1.33, allowing for a threshold quality of 30% and a minimal read length of 20 base pairs. Reads that met the preset quality criteria were aligned to human genome GRCh38 (retrieved from the gencode database, downloaded on 1 December 2018) with STAR RNA-seq aligner version 2.7 [70]. The sequencing counts were calculated by the STAR script. In order to estimate transcriptional changes, we utilized the Degust RNA-Seq analysis platform with default parameters (originally designed by D.R. Powell) [71]. In the current study, transcripts exhibiting a log_2_ fold change greater than or equal to an absolute value of 1.585 (representing a change of threefold or more) and a false discovery rate (FDR) of less than 0.05 were considered differentially expressed.

### 4.9. Determination of Alkaline Phosphatase Activity, Osteocalcin and Calcification

The activity of ALP was assessed using cell lysates based on the hydrolysis of para-nitrophenylphosphate (p-NPP) hydrolysis [66,68]. A total of 100 µL of p-NPP (4 µg/µL) was added to cell lysates (100 µL) in a 96-well plate. The plates were incubated for 30 min at 37 °C. The enzymatic reaction was stopped with 2 M NaOH solution. A standard developed with 0 to 10 IU/mL of ALP (Thermo Scientific, Waltham, MA, USA) was used to calculate the activity of ALP in the tested samples, given in international units (IU). The absorbance was measured at 405 nm (Multiskan EX reader, Thermo Scientific, Waltham, MA, USA).

The concentration of OC was determined using specific enzyme-linked immunosorbent assay (ELISA) kits (R&D Systems, Minneapolis, MN, USA), as recommended by the manufacturer [66,68].

The wells of a 96-well half-area plate (Greiner Bio-One GmbH, Kremsmünster, Austria) were coated overnight at room temperature with cytokine-specific capture antibodies in PBS, then washed three times in washing buffer (PBS/0.05% Tween 20) and blocked for 2 h with 5% Tween 20 in PBS. Next, the wells were washed again, and the tested supernatants or serial dilutions of the reference recombinant human OC (50 µL) were added, and the plate was incubated overnight at 4 °C. Then, the wells were washed and incubated for 2 h with a biotinylated monoclonal antibody diluted at 1:60 in 5% Tween 20/PBS. After the washing, an enzyme streptavidin–horseradish peroxidase solution (diluted 1:40) was added for 20 min at room temperature. After washing, a mixture (1:1) of tetra-methylbenzidine (TMB) and hydrogen peroxide was applied. The colorimetric reaction was stopped with 1 M H_2_SO_4_, and the absorbance was measured at 450 nm using a Multiskan EX reader (Thermo Scientific, Waltham, MA, USA).

Alizarin red (sigma Aldrich, Darmstad, Germany) staining was used to evaluate the mineralization process in cell cultures of osteoblasts, as previously described [72].

The cells (5 × 10^5^/mL) were cultured in osteogenic medium with or without PyoM_sol_ or PyoM_insol_ for 24 days at 39 °C with 5% CO_2_. The cells were then washed with PBS and fixed with 4% formalin for 20 min and washed thrice again with PBS. Next, the cells were stained with 4% alizarin adjusted to pH 4.1 for 30 min at room temperature and then washed 4 times with deionized water. The imaging of the stained mineralized extracellular matrix of the osteoblasts was performed under an inverted-phase contrast microscope (Motic AE2000, Xiamen, China). Calcium deposits were assessed quantitatively as follows: acetic acid (10%) was added to each well (500 µL), and the plates were incubated at room temperature for 30 min with shaking (100 rpm). The osteoblasts were detached and transferred with 10% acetic acid to 1.5 mL microcentrifuge tubes. The samples were heated to 80 °C for 10 min and then cooled on ice for 5 min. After centrifugation, the supernatants were transferred to new tubes and neutralized using 10% ammonium hydroxide. The absorbance values were measured at 405 nm (Multiskan EX, ThermoFisher, Waltham, MA, USA). The number of calcium deposits was assessed using a standard curve developed with hydroxyapatite (Sigma Aldrich, Darmstadt, Germany).

### 4.10. Cytokine Release Profile Characterization

The concentration of interleukin IL-6, IL-10, and TNF-α in the osteogenic culture media of osteoblasts incubated for 4, 7, 11, 14, 18, 21, 25, and 28 days with PyoM_sol_ or PyoM_insol_ was determined using specific enzyme-linked immunosorbent assay (ELISA) kits (R&D Systems, Minneapolis, MN, USA), as recommended by the manufacturer. The wells of 96-well half-area plate (Greiner Bio-One GmbH, Kremsmünster, Austria) were coated overnight at room temperature with cytokine-specific capture antibodies in PBS, then washed three times in washing buffer (PBS/0.05% Tween 20) and blocked for 1.5 h with PBS with 1% bovine serum albumin (BSA). After washing the wells, the tested cell culture supernatants or serial dilutions of the reference recombinant human cytokines (50 µL), used for establishing the standard curve, were added and incubated overnight at 4 °C. Then, the wells were washed and incubated for 2 h with a biotinylated monoclonal antibody diluted at 1:60 in PBS/1% BSA. Following washing, an enzyme streptavidin–horseradish peroxidase solution (diluted 1:40) was added for 20 min at room temperature. After washing, a mixture (1:1) of tetramethylbenzidine (TMB) and hydrogen peroxide was applied. The colorimetric reaction was stopped with 1 M H_2_SO_4_. The absorbance was measured at 450 nm using a Multiskan EX reader (Thermo Scientific, Waltham, MA, USA).

### 4.11. Antibacterial Activity of PyoM_sol_ and PyoM_insol_

The antibacterial activity of PyoM_sol_ and PyoM_insol_ was determined against the reference *Staphylococcus aureus* ATCC 29,213 and two clinical strains isolated from bone infection, namely, *S. aureus* resistant to methicillin (MRSA) and *S. felis*. The minimum inhibitory concentration (MIC) was evaluated using a resazurin reduction assay [73]. The bacteria were cultured in Mueller–Hinton Broth (MHB) to the mid-log phase, and the inoculum was standardized to 5 × 10^5^ CFU/mL, as recommended by European Committee on Antimicrobial Susceptibility Testing (EUCAST) guidelines [74,75]. PyoM_sol_ or PyoM_insol_ were added to the wells of a 96-well plate (Nunc, Rochester, NY, USA) with 100 μL MHB. PyoM were diluted (2-fold dilutions) at a concentration ranging from 1 to 1024 μg/mL. After the addition of the bacterial suspension (100 μL/well), the plates were incubated for 24 h at 37 °C. The control wells contained the bacterial suspension without the tested PyoM (positive control of bacterial growth). Additionally, wells with the bacterial medium only (negative control) were included. To each well, 20 μL of 0.02% resazurin solution (Sigma Aldrich, Darmstadt, Germany) in sterile PBS was added for 3 h. The fluorescence was measured at 560 nm (excitation) and 590 nm (emission) (SpectraMax^®^ i3x Multi-Mode Microplate Reader, Molecular Devices, San Jose, CA, USA).

### 4.12. Statistical Analysis

GraphPad Prism version 9.1.0 for Windows (GraphPad Software, San Diego, CA, USA) was used to perform the statistical analyses and create the graphs. ANOVA with Dunnett’s post hoc test was used to compare the data.

## 5. Conclusions

Recently, many innovative biomolecules have been tested in order to create novel bone regeneration strategies. The “perfect biomolecule” for the regeneration of bone defects would support not only the growth and differentiation of osteoblasts, minimizing cell apoptosis, but also reduce the risk of bacterial infections. A novel approach seems to be the controlled imitation of pathological processes dependent on the deposition of HGA polymers occurring in AKU to enhance the osteoinduction, mineralization, and regeneration of bone tissue. The data presented in this study indicate that polymeric PyoM isolated from *P. aeruginosa* is cytocompatible with osteoblasts, and it promotes cell survival and differentiation in conjunction with the limitation of cell apoptosis. The results presented in this study lead to considering PyoM as a stimulator of osteoinductive processes and osteoblast maturation. The stimulation of osteoblasts with PyoM provided appropriate conditions to promote in vitro bone regeneration by increasing the cell migration and production of essential ossification markers, including ALP, OC, and increased calcification. Moreover, PyoM stimulated osteoblasts to increase the production of IL-6, IL-10, and TNF-α, which are involved in osteogenesis. Although both the PyoM variants displayed quite similar properties, PyoM_sol_ appears to have greater osteoinductive and pro-regenerative potential due to the upregulation of cell proliferation. PyoM_sol_ showed a weaker ability to stimulate the production of pro-inflammatory TNF-α by osteoblasts. This difference may also promote PyoM_sol_ over PyoM_insol_ due to it diminishing the risk of a deleterious inflammatory response as a result of medical intervention. The activation of osteoblasts with PyoM presents an opportunity to develop PyoM-modified biocomposites for potential therapeutic applications in regenerative medicine. The antibacterial properties of PyoM reinforce this idea.

However, further research is needed to deeply explain the mechanism of action of PyoM and to determine whether or not PyoM affects osteoblast/osteoclast homeostasis.

## Data Availability

The data generated during this study are available at the University of Lodz, Faculty of Biology and Environmental Protection, Department of Immunology and Infectious Biology, Łódź, 90-237, Poland, and they are available from the corresponding authors upon request.

## Supplementary Materials

The following supporting information can be downloaded at: https://www.mdpi.com/article/10.3390/ijms252413406/s1.

## References

[1] Ansari M. (2019). Bone tissue regeneration: Biology, strategies and interface studies. Prog. Biomater..

[2] Florencio-Silva R., Sasso G.R.D.S., Sasso-Cerri E., Simões M.J., Cerri P.S. (2015). Biology of bone tissue: Structure, function, and factors that influence bone cells. BioMed Res. Int..

[3] Blair H.C., Larrouture Q.C., Li Y., Lin H., Beer-Stoltz D., Liu L., Tuan R.S., Robinson L.J., Schlesinger P.H., Nelson D.J. (2017). Osteoblast differentiation and bone matrix formation in vivo and in vitro, Tissue. Eng. Part B Rev..

[4] Crockett J.C., Rogers M.J., Coxon F.P., Hocking L.J., Helfrich M.H. (2011). Bone remodelling at a glance. J. Cell Sci..

[5] Langdahl B., Ferrari S., Dempster D.W. (2016). Bone modeling and remodeling: Potential as therapeutic targets for the treatment of osteoporosis. Ther. Adv. Musculoskelet. Dis..

[6] Noh J.Y., Yang Y., Jung H. (2020). Molecular mechanisms and emerging therapeutics for osteoporosis. Int. J. Mol. Sci..

[7] Szulc P. (2018). Bone turnover: Biology and assessment tools. Best Pract. Res. Clin. Endocrinol. Metab..

[8] Ono T., Hayashi M., Sasaki F., Nakashima T. (2020). RANKL biology: Bone metabolism, the immune system, and beyond. Inflamm. Regen..

[9] Xu J., Yu L., Liu F., Wan L., Deng Z. (2023). The effect of cytokines on osteoblasts and osteoclasts in bone remodeling in osteoporosis: A review. Front. Immunol..

[10] TKatagiri, Watabe T. (2016). Bone morphogenetic proteins. Cold Spring Harb. Perspect. Biol..

[11] Lin X., Patil S., Gao Y.G., Qian A. (2020). The bone extracellular matrix in bone formation and regeneration. Front. Pharmacol..

[12] Shuaishuai W., Tongtong Z., Dapeng W., Mingran Z., Xukai W., Yue Y., Hengliang D., Guangzhi W., Minglei Z. (2023). Implantable biomedical materials for treatment of bone infection. Front. Bioeng. Biotechnol..

[13] Li J., Wong R.M.Y., Chung Y.L., Leung S.S.Y., Chow K.-H., Ip M., Cheung W.-H., Man R., Wong Y. (2022). Fracture-related infection in osteoporotic bone causes more severe infection and further delays healing. Bone Joint. Res..

[14] Croes M., van der Wal B.C.H., Vogely H.C. (2019). Impact of bacterial infections on osteogenesis: Evidence from in vivo studies. J. Orthop. Res..

[15] Taylor A.M., Hsueh M.F., Ranganath L.R., Gallagher J.A., Dillon J.P., Huebner J.L., Catterall J.B., Kraus V.B. (2017). Cartilage biomarkers in the osteoarthropathy of alkaptonuria reveal low turnover and accelerated ageing. Rheumatology.

[16] Davison A.S., Hughes A.T., Milan A.M., Sireau N., Gallagher J.A., Ranganath L.R. (2020). Alkaptonuria—Many questions answered, further challenges beckon. Ann. Clin. Biochem..

[17] Chow Y., Norman B.P., Roberts N.B., Ranganath L.R., Teutloff C., Bittl R., Duer M.J., Gallagher J.A., Oschkinat H. (2020). Pigmentation chemistry and radical-based collagen degradation in alkaptonuria and osteoarthritic cartilage. Angew. Chem. Int. Ed. Engl..

[18] Roberts N.B., Curtis S.A., Milan A.M., Ranganath L.R. (2015). The pigment in alkaptonuria relationship to melanin and other coloured substances: A review of metabolism, composition and chemical analysis. JIMD Rep..

[19] Nadzir M.M., Nurhayati R.W., Idris F.N., Nguyen M.H. (2021). Biomedical applications of bacterial exopolysaccharides: A review. Polymers.

[20] Farshidfar N., Iravani S., Varma R.S. (2023). Alginate-based biomaterials in tissue engineering and regenerative medicine. Mar. Drugs..

[21] Lorquin F., Piccerelle P., Orneto C., Robin M., Lorquin J. (2022). New insights and advances on pyomelanin production: From microbial synthesis to applications. J. Ind. Microbiol. Biotechnol..

[22] Behzadi P., Baráth Z., Gajdács M. (2021). It’s not easy being green: A narrative review on the microbiology, virulence and therapeutic prospects of multidrug-resistant Pseudomonas aeruginosa. Antibiotics.

[23] Urbaniak M.M., Gazińska M., Rudnicka K., Płociński P., Nowak M., Chmiela M. (2023). In vitro and in vivo biocompatibility of natural and synthetic Pseudomonas aeruginosa pyomelanin for potential biomedical applications. Int. J. Mol. Sci..

[24] Lorquin F., Ziarelli F., Amouric A., Di Giorgio C., Robin M., Piccerelle P., Lorquin J. (2021). Production and properties of non-cytotoxic pyomelanin by laccase and comparison to bacterial and synthetic pigments. Sci. Rep..

[25] Roy S., Rhim J.W. (2022). New insight into melanin for food packaging and biotechnology applications. Crit. Rev. Food Sci. Nutr..

[26] Urbaniak M.M., Rudnicka K., Gościniak G., Chmiela M. (2023). Can pyomelanin produced by Pseudomonas aeruginosa promote the regeneration of gastric epithelial cells and enhance Helicobacter pylori phagocytosis?. Int. J. Mol. Sci..

[27] ElObeid A.S., Kamal-Eldin A., Abdelhalim M.A.K., Haseeb A.M. (2017). Pharmacological properties of melanin and its function in health. Basic Clin. Pharmacol. Toxicol..

[28] Liu D., Zhong Z., Karin M. (2022). NF-κB: A double-edged sword controlling inflammation. Biomedicines.

[29] (2009). Biological Evaluation of Medical Devices—Part 5: Tests for in Vitro Cytotoxicity.

[30] Ge S.X., Jung D., Yao R. (2020). ShinyGO: A graphical enrichment tool for animals and plants. Bioinformatics.

[31] Blumer M.J.F. (2021). Bone tissue and histological and molecular events during development of the long bones. Ann. Anat..

[32] Berendsen A.D., Olsen B.R. (2015). Bone development. Bone.

[33] Wu A.M., Bisignano C., James S.L., Abady G.G., Abedi A., Abu-Gharbieh E., Alhassan R.K., Alipour V., Arabloo J., Asaad M. (2021). Global, regional, and national burden of bone fractures in 204 countries and territories, 1990–2019: A systematic analysis from the Global Burden of Disease Study 2019. Lancet Healthy Longev..

[34] Kemmak A.R., Rezapour A., Jahangiri R., Nikjoo S., Farabi H., Soleimanpour S. (2020). Economic burden of osteoporosis in the world: A systematic review. Med. J. Islam. Repub. Iran..

[35] Szwed-Georgiou A., Płociński P., Kupikowska-Stobba B., Urbaniak M.M., Rusek-Wala P., Szustakiewicz K., Piszko P., Krupa A., Biernat M., Gazińska M. (2023). Bioactive materials for bone regeneration: Biomolecules and delivery systems. ACS Biomater. Sci. Eng..

[36] Weycker D., Li X., Barron R., Bornheimer R., Chandler D. (2016). Hospitalizations for osteoporosis-related fractures: Economic costs and clinical outcomes. Bone. Rep..

[37] Ferraz A.R., Pacheco R., Vaz P.D., Pintado C.S., Ascensão L., Serralheiro M.L. (2021). Melanin: Production from cheese bacteria, chemical characterization, and biological activities. Int. J. Environ. Res. Public Health.

[38] Kurian N.K., Bhat S.G. (2018). Data on the characterization of non-cytotoxic pyomelanin produced by marine Pseudomonas stutzeri BTCZ10 with cosmetological importance. Data Brief.

[39] Amarasekara D.S., Kim S., Rho J. (2021). Regulation of osteoblast differentiation by cytokine networks. Int. J. Mol. Sci..

[40] Jilka R.L., Weinstein R.S., Bellido T., Roberson P., Parfitt A.M., Manolagas S.C. (1999). Increased bone formation by prevention of osteoblast apoptosis with parathyroid hormone. J. Clin. Investig..

[41] Ren R., Guo J., Chen Y., Zhang Y., Chen L., Xiong W. (2021). The role of Ca2+/calcineurin/NFAT signalling pathway in osteoblastogenesis. Cell Prolif..

[42] Zayzafoon M. (2006). Calcium/calmodulin signaling controls osteoblast growth and differentiation. J. Cell Biochem..

[43] Gao S., Chen B., Zhu Z., Du C., Zou J., Yang Y., Huang W., Liao J. (2023). PI3K-Akt signaling regulates BMP2-induced osteogenic differentiation of mesenchymal stem cells (MSCs): A transcriptomic landscape analysis. Stem. Cell. Res..

[44] Vimalraj S. (2020). Alkaline phosphatase: Structure, expression and its function in bone mineralization. Gene.

[45] Le-Vinh B., Akkuş-Dağdeviren Z.B., Le N.M.N., Nazir I., Bernkop-Schnürch A. (2022). Alkaline phosphatase: A reliable endogenous partner for drug delivery and diagnostics. Adv. Ther..

[46] Moser S.C., van der Eerden B.C.J. (2019). Osteocalcin—A versatile bone-derived hormone. Front. Endocrinol..

[47] Zoch M.L., Clemens T.L., Riddle R.C. (2016). New insights into the biology of osteocalcin. Bone.

[48] Jilka R.L., Weinstein R.S., Bellido T., Parfitt A.M., Manolagas S.C. (1998). Osteoblast programmed cell death (apoptosis): Modulation by growth factors and cytokines. J. Bone Miner. Res..

[49] Blanchard F., Duplomb L., Baud’huin M., Brounais B. (2009). The dual role of IL-6-type cytokines on bone remodeling and bone tumors. Cytokine Growth Factor Rev..

[50] Itoh S., Udagawa N., Takahashi N., Yoshitake F., Narita H., Ebisu S., Ishihara K. (2006). A critical role for interleukin-6 family-mediated Stat3 activation in osteoblast differentiation and bone formation. Bone.

[51] Yoshitake F., Itoh S., Narita H., Ishihara K., Ebisu S. (2008). Interleukin-6 directly inhibits osteoclast differentiation by suppressing receptor activator of NF-κB signaling pathways. J. Biol. Chem..

[52] Chen E., Liu G., Zhou X., Zhang W., Wang C., Hu D., Xue D., Pan Z. (2018). Concentration-dependent, dual roles of IL-10 in the osteogenesis of human BMSCs via P38/MAPK and NF-kB signaling pathways. FASEB J..

[53] Liu D., Yao S., Wise G.E. (2006). Effect of Interleukin-10 on gene expression of osteoclastogenic regulatory molecules in the rat dental follicle. Eur. J. Oral Sci..

[54] Xin L.X., Kukita T., Kukita A., Otsuka T., Niho Y., Iijima T. (1995). Interleukin-10 selectively inhibits osteoclastogenesis by inhibiting differentiation of osteoclast progenitors into preosteoclast-like cells in rat bone marrow culture system. J. Cell Physiol..

[55] Lee K., Seo I., Choi M.H., Jeong D. (2018). Roles of mitogen-activated protein kinases in osteoclast biology. Int. J. Mol. Sci..

[56] Dresner-Pollak R., Gelb N., Rachmilewitz D., Karmeli F., Weinreb M. (2004). Interleukin 10-deficient mice develop osteopenia, decreased bone formation, and mechanical fragility of long bones. Gastroenterology.

[57] Claudino M., Garlet T.P., Cardoso C.R.B., De Assis G.F., Taga R., Cunha F.Q., Silva J.S., Garlet G.P. (2010). Down-regulation of expression of osteoblast and osteocyte markers in periodontal tissues associated with the spontaneous alveolar bone loss of interleukin-10 knockout mice. Eur. J. Oral Sci..

[58] Kobayashi K., Takahashi N., Jimi E., Udagawa N., Takami M., Kotake S., Nakagawa N., Kinosaki M., Yamaguchi K., Shima N. (2000). Suda, tumor necrosis factor stimulates osteoclast differentiation by a mechanism independent of the ODF/RANKL-RANK interaction. J. Exp. Med..

[59] Osta B., Benedetti G., Miossec P. (2014). Classical and paradoxical effects of TNF-α on bone homeostasis. Front. Immunol..

[60] Kitaura H., Marahleh A., Ohori F., Noguchi T., Nara Y., Pramusita A., Kinjo R., Ma J., Kanou K., Mizoguchi I. (2022). Role of the interaction of tumor necrosis factor-α and tumor necrosis factor receptors 1 and 2 in bone-related cells. Int. J. Mol. Sci..

[61] Marcello E., Maqbool M., Nigmatullin R., Cresswell M., Jackson P.R., Basnett P., Knowles J.C., Boccaccini A.R., Roy I. (2021). Antibacterial composite materials based on the combination of polyhydroxyalkanoates with selenium and strontium co-substituted hydroxyapatite for bone regeneration. Front. Bioeng. Biotechnol..

[62] Zerrad A., Anissi J., Ghanam J., Sendide K., El Hassouni M. (2014). Antioxidant and antimicrobial activities of melanin produced by a Pseudomonas balearica strain. J. Biotechnol. Lett..

[63] Xu C., Li J., Yang L., Shi F., Yang L., Ye M. (2017). Antibacterial activity and a membrane damage mechanism of Lachnum YM30 melanin against *Vibrio parahaemolyticus* and *Staphylococcus aureus*. Food Control.

[64] El-Naggar N.E.A., Saber W.I.A. (2022). Natural melanin: Current trends, and future approaches, with especial reference to microbial source. Polymers.

[65] Schiavone M.L., Millucci L., Bernardini G., Giustarini D., Rossi R., Marzocchi B., Santucci A. (2020). Homogentisic acid affects human osteoblastic functionality by oxidative stress and alteration of the Wnt/β-catenin signaling pathway. J. Cell Physiol..

[66] Kurzyk A., Szwed-Georgiou A., Pagacz J., Antosik A., Tymowicz-Grzyb P., Gerle A., Szterner P., Włodarczyk M., Płociński P., Urbaniak M.M. (2023). Calcination and ion substitution improve physicochemical and biological properties of nanohydroxyapatite for bone tissue engineering applications. Sci. Rep..

[67] Mnich E., Kowalewicz-Kulbat M., Sicinska P., Hinc K., Obuchowski M., Gajewski A., Moran A.P., Chmiela M. (2016). Impact of Helicobacter pylori on the healing process of the gastric barrier. World J. Gastroenterol..

[68] Piszko P., Włodarczyk M., Zielińska S., Gazińska M., Płociński P., Rudnicka K., Szwed A., Krupa A., Grzymajło M., Sobczak-Kupiec A. (2021). PGS/HAp microporous composite scaffold obtained in the TIPS-TCL-SL method: An innovation for bone tissue engineering. Int. J. Mol. Sci..

[69] Kawka M., Płocińska R., Płociński P., Pawełczyk J., Słomka M., Gatkowska J., Dzitko K., Dziadek B., Dziadek J. (2023). The functional response of human monocyte-derived macrophages to serum amyloid A and Mycobacterium tuberculosis infection. Front. Immunol..

[70] Dobin A., Davis C.A., Schlesinger F., Drenkow J., Zaleski C., Jha S., Batut P., Chaisson M., Gingeras T.R. (2013). STAR: Ultrafast universal RNA-seq aligner. Bioinformatics.

[71] Powell D. Degust: Interactive RNA-Seq Analysis. http://degust.erc.monash.edu.

[72] Prasadh S., Gupta M., Wong R. (2022). In vitro cytotoxicity and osteogenic potential of quaternary Mg-2Zn-1Ca/X-Mn alloys for craniofacial reconstruction. Sci. Rep..

[73] Sandberg M.E., Schellmann D., Brunhofer G., Erker T., Busygin I., Leino R., Vuorela P.M., Fallarero A. (2009). Pros and cons of using resazurin staining for quantification of viable Staphylococcus aureus biofilms in a screening assay. J. Microbiol. Methods.

[74] European Committee on Antimicrobial Susceptibility Testing (2023). Breakpoint Tables for Interpretation of MICs and Zone Diameters.

[75] Słota D., Piętak K., Florkiewicz W., Jampílek J., Tomala A., Urbaniak M.M., Tomaszewska A., Rudnicka K., Sobczak-Kupiec A. (2023). Clindamycin-loaded nanosized calcium phosphates powders as a carrier of active substances. Nanomaterials.

